# A Future Avenue of Treatment Ulcerative Colitis Targeting Macrophage Polarization: A Phytochemical Application

**DOI:** 10.1093/crocol/otae070

**Published:** 2024-11-28

**Authors:** Nishant Kumar Saurabh, Mohd Mabood Khan, Annet Kirabo

**Affiliations:** Division of Molecular Biology, National Institute of Cancer Prevention & Research (ICMR-NICPR), I-7, Sector-39, Noida 201301, India; Department of Medicine, Robinson Research Building, Vanderbilt University Medical Centre, Nashville, TN 37232-6602, USA; Department of Medicine, Robinson Research Building, Vanderbilt University Medical Centre, Nashville, TN 37232-6602, USA

**Keywords:** phytochemical, macrophage, ulcerative colitis, polarization

## Abstract

**Background:**

Ulcerative colitis (UC) is a prevalent inflammatory bowel disease primarily impacting the mucosa of the colon. It is characterized by recurring and incurable symptoms and causes immense suffering and significant economic burden due to limited treatment options. Typical symptoms of UC include diarrhea, alterations in bowel patterns, bleeding from the rectum, rectal pain or urgency, anemia, and tiredness. Therefore, developing novel and effective treatment strategies for UC is imperative.

**Purpose:**

This review aimed to explain how macrophage polarization contributes to UC development and compiled information on natural compounds with promising therapeutic potential that can target the macrophage phenotype and shed light on its potential mode of action.

**Results:**

The phenotypic alteration of macrophages profoundly affects the development of UC, and these cells are essential for preserving intestinal immunological homeostasis. Evidence from research suggests that one effective method for UC prevention and therapy is to guide macrophage polarization toward the M2 phenotype. Phytochemicals, which are compounds extracted from plants, possess a wide array of biological activities. For example: Ginsenoside Rg1 emerges as a crucial regulator of macrophage polarization, promoting the M2 phenotype while inhibiting the M1 phenotype. Notably, their low toxicity and high effectiveness render them promising candidates for therapeutic interventions. These compounds have demonstrated encouraging protective effects against inflammation in the colon.

**Conclusions:**

Exploring phytochemicals as a therapeutic avenue targeting macrophage polarization presents an innovative approach to treating UC.

## Introduction

Ulcerative colitis (UC) is an inflammatory bowel disease (IBD) affecting both males and females. Chronic, inflammatory UC has no known cause and is characterized by recurring and incurable symptoms. Statistics show that the occurrence of UC has increased dramatically over the last few decades, with rates varying from 0.5 to 31.5 per 100 000 people per year among different populations.^1,2^ The global incidence of this disease has risen significantly as a result of countries’ industrialization,^3^ and it is continuing to rise at an increasing rate due to peoples’ diets, lifestyles, and environmental factors like geography and living circumstances.^4^ The ages range 15-39 years are the most common for this disease, however, the condition affects both sexes equally. Genetics,^5,6^ environment,^7^ and the microbiome^8^ are some of the factors that contribute to UC, which can affect the mucosa of the colon and rectum consecutively. As a result, the observable manifestations vary depending on which part of the gastrointestinal system is affected.^9,10^ Ulcerative colitis is characterized by continuing stomach pain, bloody stools, diarrhea, altered bowel habits, fever, anemia, fatigue, and incontinence.^11,12^ The first thing you should consider is the infectious agents of inflammation like Salmonella, Shigella, Yersinia, Campylobacter, Aeromonas, *Escherichia coli*, and amebae that cause the typical symptoms of the UC. In relation to rectal bleeding, factors such as anal fissures, hemorrhoids, diverticula, and polyps should also be taken into consideration.^13^

Accurately identifying the affected body part allows for the most effective treatment approach. On the other hand, distinguishing between UC and Crohn’s disease is useful when selecting a course of treatment and surgical procedures. Indeterminate colitis occurs when it is difficult to distinguish between these two disorders in some circumstances. Currently, there is no “gold standard” test for UC.^14^ Colonoscopy, histology, blood tests, fecal examination, endoscopy, and radiographic examinations are the mainstays of the diagnostic process for UC.^15^ A meta-analysis of cohort research found that individuals with UC had a 2.4-fold increased risk of colorectal cancer and that this risk was higher in males compared to females and in patients diagnosed with UC and severe colitis at a younger age.^16^

The inflammatory pathway in colitis increases myeloperoxidase (MPO), inducible nitric oxide (NO) synthase, and cox-2 levels, leading to decreased anti-inflammatory proteins and increased pro-inflammatory proteins like IL-6, IL-1B, and TNF-a. This results in oxidative stress, inflammation, and antioxidant decline, causing cell inflammation, immune cell invasion, lining damage, and intestinal barrier disruption.^17^

Ulcerative colitis is typically treated with immunosuppressive and anti-inflammatory medications. Among the drugs used in mild to severe levels of the disease can be a family of aminosalicylates such as sulfalazine,^18^ pentase,^19^ Asacol,^20^ balsalazide,^21^ and budesonide^22^ from the category of corticosteroids and azathioprine, mercaptopurine,^23^ Methotrexate,^24^ cyclosporine A,^25^ infliximab, sertolizumab, adalimumab.^26^ Hepatitis, pancreatitis, diabetes mellitus, hypertension, hyperlipidemia, osteoporosis, and hemolytic anemia are only some of the severe adverse effects of these medicines.^18^ The severe side effects and inadequate efficiency of these medications, however, present a significant obstacle to efficient treatment. Additionally, there at present no effective treatments for UC that can make up for the drawbacks of standard care.^27^

Natural remedies are currently a promising alternative to traditional medicines for the treatment of UC. A class of active polyphenolic chemicals known as flavonoids, having 2-phenylchromones as their fundamental building block, can be found in plants. Natural herbal remedies with flavonoids provide beneficial anti-inflammatory, antioxidant, and cardiovascular disease therapy properties with few side effects and good safety.^28^

Therefore, the focus of this study is on the different kinds of flavonoid herbal natural products’ mechanisms of action in UC. Herbal medicines have been used to treat UC in recent years and have been successful in clinical applications. This review the state-of-the-art information on herbal therapy and traditional Iranian and Chinese medicine for the therapy of UC patients and talks about recent developments in their function as disease preventatives.

## The Pathogenesis of UC

**Figure 1.**
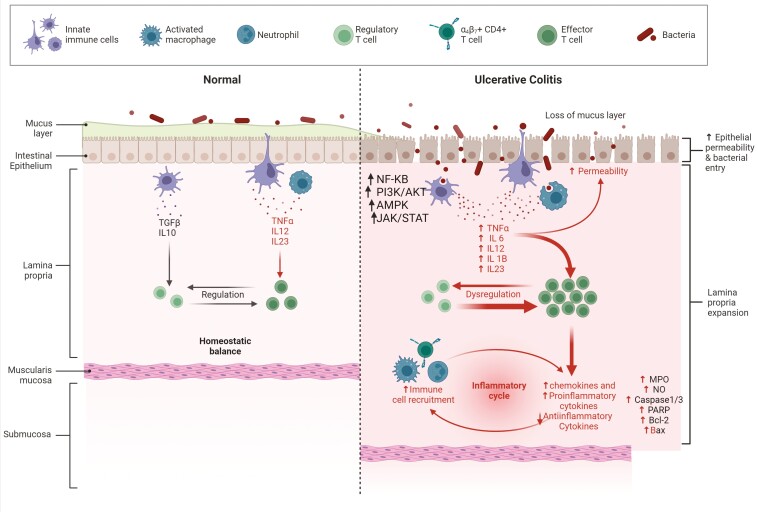
The major events in the pathogenesis of ulcerative colitis.

The pathogenesis of UC involves a complex interplay of factors contributing to the development and progression of this IBD. Ulcerative colitis’s pathophysiology is primarily characterized by a compromised intestinal barrier, immunological response, and intestinal flora, as depicted in Figure 1.

### Immune Response

Understanding UC pathophysiology requires understanding the immune system and inflammatory mechanisms. Dendritic cells (DCs), macrophages, and neutrophils are linked to innate immune response. Elevated production of proteins like neutrophil elastase and myeloperoxidase is observed in UC.^29–31^ Dendritic cells and macrophages present, take up, and digest antigens as part of the immunological response. Pro-inflammatory substances like TNF-a and IL-6 can be released when they activate signaling pathways including NF-kB, PI3K/AKT, AMPK, and JAK/STAT. In addition to innate immunity, adaptive immunity is made up mostly of T cells and is highly specialized. Helper T cells are capable of activation and differentiation into Th1, Th2, or Th17 cells, which help eliminate certain infections from the body. However, aberrant T-cell development has been linked to UC and Crohn’s disease because it can cause inflammation.^32^ By IL-10 and TGF-b production, Th0 cell suppression, and control of gut immunological responses, regulatory T cells (Tregs) manage mucosal homeostasis. Through inflammatory pathways, cytokines also function as messenger molecules that promote UC development.^31^

### Cytokines

Cytokines are inflammatory mediators present in intestinal mucosa that aid in the progression of UC. Pro-inflammatory and anti-inflammatory cytokines can be distinguished between them. Dysregulation of the pro-anti-inflammatory cytokine balance as a preponderance of pro-inflammatory cytokine production is associated with the pathophysiology of UC. Colonic epithelium destruction, crypt abscesses, and small-vessel vasculitis are all caused by the aggravation of intestinal diseases brought on by enhanced cytokines that induce inflammation and neutrophil buildup.^33^ The pathophysiology of UC is strongly linked to the increased expression of pro-inflammatory molecules and the reduced expression of anti-inflammatory ones, making it crucial to suppress pro-inflammatory production.

### Oxidative Stress

Inflammation-related damage, including excessive oxidative stress and cytokine release, is a significant pathogenic process in UC. This leads to a loss of mucosal barrier function and decreased tight junction proteins, increasing the risk of developing UC. Colons produce oxidative metabolites, disrupting mucosal pH balance and accelerating intestinal epithelial cell (IEC) apoptosis. Increased MPO and NO levels also infiltrate inflammatory cells in UC colonic tissue.^34–36^

### Apoptosis

Colonic crypt cells use apoptosis to eliminate senescent cells, preserving intestinal functional homeostasis. In UC patients, a higher rate of colonic mucosal epithelial cell death leads to structural damage to crypt cells, damaging the intestinal mucosal mechanical barrier. Apoptosis-defining proteins include Bcl-2, PARP, Bax, and caspase-1/3, and dextran sodium sulfate (DSS)-induced colitis can increase these concentrations.^37–40^

## Macrophage

Intestinal macrophages are an essential part of innate immunity and are polarized to different symptoms by environmental cues. They perform a variety of diverse tasks, including pathogen recognition, phagocytosis of microbes and debris, remodeling of damaged tissues, support of regulatory T cells, and inflammation control. These actions are thought to be the primary contributors to and maintainers of intestinal homeostasis.^41–43^

By creating in vitro macrophage models from monocytes, it is possible to distinguish between two subsets of the population that have diametrically opposed roles: the classically activated (M1) macrophages, which represent pro-inflammatory conditions, and the alternatively activated (M2) macrophages, which represent anti-inflammatory conditions.^44–48^ Inflammatory disorders may develop if the equilibrium of macrophage polarization is upset, limiting the body’s capacity to maintain stability and detect indications of tissue injury.^49^ Researchers have found that when certain cytokines or complexes, like TNF-α, LPS, TGF-β, IL-10, IL-13, Glucocorticoids, or the immune complex, activate macrophages, they go through a range of activation states. Along the M1/M2 axis, these activation states are similar but different in terms of transcriptional and functional processes.^50–52^ However, macrophage polarization into M1 or M2 helps us understand their diverse activities and transformations.

One of the studies conducted by Li et al.^53^ used Human monocyte leukemic cells (THP-10) cell lines and were cultured with 100 ng/mL phorbol 12-mysistate 12-acetate (PMA; Sigma-Aldrich) for 24 hours to induce macrophage differentiation, as previously described. Afterward, the M1 subtype of macrophages was inducible by treating them with 100 ng/mL of lipopolysaccharide (LPS; Sigma-Aldrich) and 40 ng/mL of interferon-g (IFN-g; Sigma-Aldrich) for a time period of 24 hours.^54^ Additionally, M0 macrophages produced by THP-1 were exposed to 20 ng/mL IL-4 (PeproTech) and 20 ng/mL IL-13 (PeproTech) for a duration of 24 hours in order to bring about M2 macrophage polarization.^55^

### M1 Macrophage

M1 macrophages are activated by various factors including tumor necrosis factor-a (TNF-a), toll-like receptor (TLR) ligands, lipopolysaccharides, IFN-g, and pro-inflammatory cytokines. They can introduce antigens, eradicate infections, and generate resistance. They produce NO, which prevents tissue damage from reactive oxygen species, inhibits tissue regeneration, and aids wound healing. M1 macrophages release iNOS (inducible NO synthase), a cytokine thought to be an anti-inflammatory.^56,57^

TNF-a is a powerful inflammatory mediator released by activated M1 macrophages. It has several biological effects, including cell proliferation, differentiation, and various pro-inflammatory effects. To regulate inflammation, macrophages release a cascade of chemicals after being stimulated with lipopolysaccharide (LPS). These include prostaglandins (PG), interleukin-1 (IL-1), NO, and TNF-a.^58^

### M2 Macrophage

M2 macrophages may be recognized by their distinctive markers, such as IL-10, CD206, and CD163,^59^ and they are polarized by boosting the Th2 cytokines IL-4 and IL-13 through the activation of STAT6 through the IL-4 receptor alpha (IL-4Ra). M2 macrophages are activated by IL-10 during expansion by activating STAT3 through the IL-10R (IL-10 receptor). Excellent phagocytosis, the removal of apoptotic debris, wound healing speed, and the promotion of angiogenesis and fibrosis are all features of M2 macrophages. They increase the amount of IL-10 and arginase 1, which impede secure processes.^57,60^

### The Polarization of Macrophages Regulates the Development of UC

**Figure 2.**
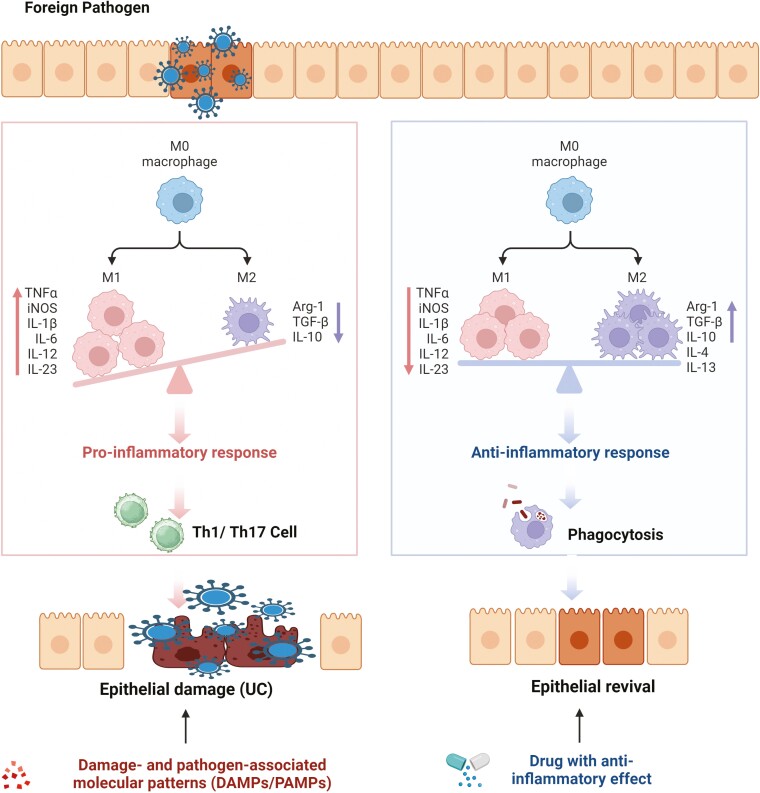
The major events of macrophage polarization in the regulation of UC. UC, ulcerative colitis.

The origin of macrophages suggests M1 and M2 macrophages are distinct subsets polarized from a common precursor, displaying diverse phenotypes and functions influenced by environmental cues.^42,61^ Ulcerative colitis is a chronic inflammatory disease caused by the disruption of the epithelial barrier, a single layer of intestinal cells that controls luminal content permeability.^62^ Foreign pathogens invade the intestinal epithelial layer, causing M1 macrophages to engulf foreign materials and secrete pro-inflammatory cytokines, promoting immune responses via Th1 and Th17 cells.^49^ The protective inflammatory response, self-limited and self-resolving after pathogen elimination, is controlled by M1 macrophage recruitment and M2 macrophage accumulation, preventing tissue damage or wound healing impairment.^63^ The infiltration of M1 macrophages disrupts macrophage polarization, leading to overexpression of inflammatory cytokines and iNOS, which directly or indirectly affects IECs, causing injury or necrosis, and elevating UC development.^64^ Inflammation and homeostasis promote macrophage polarization as shown in Figure 2.

Lissner et al.^65^ demonstrated this to directly break the epithelial barrier, M1 macrophages infiltrate the intestine, disrupt tight junction proteins, and trigger apoptosis in epithelial cells. The polarization of macrophages in the intestinal mucosa of patients with IBD and experimental colitis mice prioritizes the M1 phenotype.^65–67^ It should be highlighted that while resident M2-like macrophages exist as well to fight inflammation and promote wound healing, which aids in the resolution of the inflammation, colonic M1 macrophages prevail during colitis despite this.^68^ Furthermore, encouraging the anti-inflammatory M2 macrophage phenotype has been recognized as a possible therapy for UC.

### Macrophage Polarization as a Viable Strategy for UC Treatment

Research indicates that a promising approach to prevent and treat UC involves targeting the imbalanced axis of macrophage polarization. Studies reveal a positive correlation between the enrichment of M1 macrophages and the severity of the disease.^69^ In experiments involving mice induced with dinitrobenzene sulfonic acid, injecting bone marrow-derived M2 macrophages reduced colitis severity. Conversely, mice with deficient M2 macrophage polarization were found to be more susceptible to colitis induced by DSS.^70–72^

Additionally, a study by Caprioli et al.^73^ established a link between the downregulation of M1 macrophage pathway genes and mucosal healing in IBD patients treated with infliximab. Reduced M1 macrophages were associated with increased macrophage apoptosis, a key mechanism contributing to the success of anti-tumor necrosis factor antibody treatment. Eissa et al.^74^ provided evidence that in UC patients, pro-inflammatory macrophages infiltrate the intestinal mucosa, producing inflammatory mediators through NF-kB signaling. Chromofungin (CHR), an antimicrobial peptide downregulated in UC, demonstrated mitigating effects on colitis by reducing M1 macrophage markers. Furthermore, intracolonic treatment with CHR promoted M2 macrophage polarization, reducing colonic collagen deposition and maintaining IEC homeostasis, thus protecting against UC induced by DSS.^75^

Follistatin-like protein 1 (FSTL1), a cytokine highly expressed in human and mouse UC, was found to promote pro-inflammatory macrophages and inhibit anti-inflammatory ones, leading to excessive inflammatory cytokine production. Inhibition of FSTL1 was identified as a potential strategy for inducing UC remission.^62,76^ Moreover, to significantly reduce the severity of UC, extracellular vesicles (EVs) generated by bone marrow mesenchymal stem cells (BMSCs) could enhance M2 macrophage polarization. This enhancement was supported by an increase in CD163, the M2 marker. The JAK1/STAT1/STAT6 signaling pathway appeared to be related to this outcome.^77^ Above study’s results suggest that macrophage polarization may contribute to mucosal healing in IBD patients, highlighting its potential as a promising therapeutic target.

## Role of Phytochemicals in UC by Specifically Targeting Macrophage Polarization

**Figure 3.**
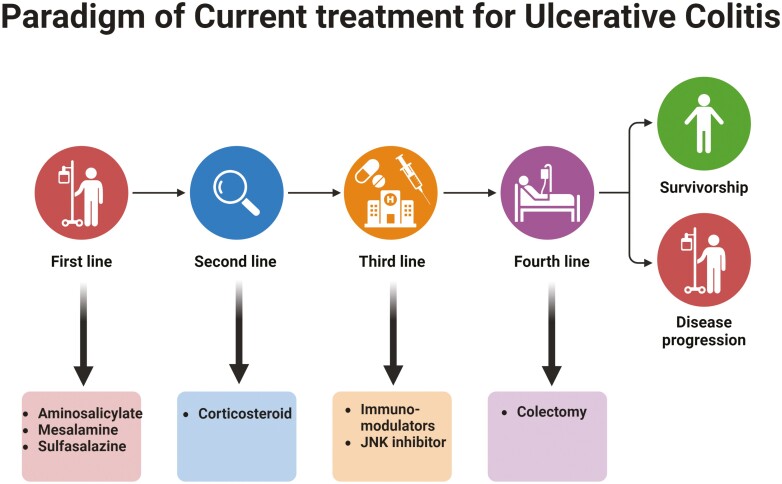
Treatment of ulcerative colitis in the current scenario.

Presently, 5-aminosalicylic acid (5-ASA) medications, available in suppositories, enemas, or oral preparations, stand as the primary first-line therapy for both severe and mild cases of UC in clinical practice.^78^ While corticosteroids can be employed in conjunction with 5-ASA medications,^79^ their use for maintaining remission is discouraged due to their short-term efficacy and potential for adverse effects.^80^ In cases of unmanageable excessive bleeding, perforation, or endoscopically inoperable unfavorable lesions associated with UC, surgery is often recommended (Figure 3).^81^

The diverse biological activities of phytochemicals, derived from readily available natural sources, have been extensively validated. Not only do they exhibit low toxicity, but they also demonstrate high effectiveness and crucial qualities in anticancer applications.^82–84^ Ongoing research is focused on identifying new phytochemical entities with fewer adverse effects for enhanced clinical applications. Recent data highlights the significant potential of active ingredients from natural products in the management of UC. This study delves into the exploration of phytochemicals targeting macrophage polarization as a therapeutic approach for treating UC (Table 1).

**Table 1.** The list of phytochemicals targeting macrophage polarization in treating ulcerative colitis.

Phytochemical	Macrophage markers	Molecular mechanism for regulating macrophage characteristics	References
GinsenosideRg1	MIF-1and PIM-1 proteins in the colon↓ CD11b^+^F4/80^+^iNOS^+^ cells in the colon↓ Arg1protein in the colon↑ CD11b^+^F4/80^+^CD206^+^ cells and CD11b^+^F4/80^+^CD163^+^cells in the colon↑	Rock1, RhoA, and Nogo-B proteins in the colon↓	^85^
Baicalin	TNF-a andIL-23 mRNA expression↓ Arg-1 and Fizz-1 mRNA expression↑ iNOS/CD206 in the colon↓ TNF-a and IL-23 mRNA expression in the colon↓ Arg-1and Fizz-1 mRNA expression in the colon↑ IRF4 protein in the colon↓ IRF5 protein in the colon↑	IRF4 siRNA inhibited iNOS/CD206 decline by baicalin	^86^
Toosendanin	CD11b^+^CD11c^+^ cells in the colon↓ F4/80^+^CD206^+^ cells in the colon↑ TNF-a, IL-6, and IL-1b levels And mRNA expression in the colon↓	NLRP3 protein↓ Nrf2 and HO-1 protein↑	^87^
Artemisinin	TNF-a, IL-1b, and IL-6 levels in the colon↓ iNOS protein↓ Arg-1protein↑ In PBMCs of CD patients CD11b^+^CD206^+^cells↑ TNF-a, IL-1b,and IL-6 levels and mRNA expression↓	ERK phosphorylation↓ MyD88 activation↓	^88^
Tiliroside	CD68^+^ iNOS^+^ cells in the colon CD68^+^ CD206^+^ cells in the colon TNF-a, IL-1b, and IL-6 mRNA expression in the colon↓ Arg-1, Chil3, and CD206 mRNA expression in the colon↑ TNF-a, IL-1b, and iNOS mRNA expression↓ Arg-1, Chil3, and CD206 mRNA expression↑ No signiﬁcant impact on Arg-1, Chil3, and CD206 mRNA expression	HIF-1a protein and mRNA expression↓	^71^
PlatycodinD	F4/80^+^iNOS^+^cells in the colon↓ F4/80^+^CD206^+^cells in the colon↑ TNF-a, IL-1b, and IL-6 levels and mRNA expression in the colon↓ IL-10 level and mRNA expression in the colon↑		^73^
Sulforaphane	F4/80^+^iNOS^+^cells↓ F4/80^+^CD206^+^cells↑ TNF-a, IL-1b, and IL-6 levels and mRNA expression↓ IL-10 level and mRNA expression↑ F4/80^+^CD68^+^cells in the colon↓ F4/80^+^CD206^+^ cells in the colon↑	p-PI3Kandp-Aktprotein↑ p-p65protein↓	^76^
Rhein	IL-1b and iNOS mRNA expression↓ IL-10 and CD206 mRNA expression↑ TNF-a, IL-1b, and IL-6 levels↓	p-STAT3protein↓	^77^
Rosmarinicacid	TNF-a, IL-1b, IL-12, NOS2,and CD16/32 mRNA expression in the colon↓ Arg, Mrc, Mgl1, and CD206 mRNA expression in the colon↑ TNF-a, IL-1b, IL-12, NOS2,and CCL4 mRNA expression↓ Arg, Mrc, Mgl1, and Dectin-1mRNA expression in the colon↑	HO-1protein↑	^79^
Genistein	CD11b^+^CD11C^+^cells in the spleen, MLNs,cLP↓ F4/80^+^CD206^+^cells in the spleen, MLNs, cLP↑ Arg1 and IL-10 levels↑ CD4% in the spleen, cLP↓ CD4% in MLNs↑ DCs in the spleen, MLNs, cLP↑ CD4^+^IL-10^+^T cells↑ in the cLP		^69^
Loganin	F4/80^+^iNOS^+^cells in the colon↓ IL-6, TNF-a, and IL-1bmRNA Expression and protein in the colon↓ MCP-1, CXCL10, and COX-2 mRNA expression in the colon↓	Sirt1 mRNA expression in the colon↑ NF-kB p65 acetylation in the colon↓	^89^
Dioscin	F4/80+CD86+ cells in the colon↓ F4/80+CD206+ cells in the colon↑ TNF-a, IFN-g, and IL-6 levels in the colon↓ IL-10 level in the colon↑ CD86 protein in the colon↓ CD206 protein in the colon↑ F4/80+CD206+ cells↑ Secretion of IL-10↑ Expression of Arg-1, IL-10, and Ym1↑ Secretion of NO, TNF-a, IL-6, and IL-1b↓ F4/80+CD86+ cells↓ Expression of iNOS, TNF-a, and IL-6↓	Glucose, lactic acid↓ Protein of Raptor, HIF- 1a, CD86, HK-2, PKM2, LDHA↓ Uptake of free fatty acids↑ Protein of CD206, ACSL1, CPT-1A, CPT-2, Rictor, PPAR-g↑	^90^
Lupeol	CD86+ cells↓ CD206+ cells↑ TNF-a and IL-1b levels↓ IL-12 and IL-10 levels↑ No significant change in CD206^+^cells and the IL-10 and IL13 levels in the colon mRNA expression of IL-12, IL-6, iNOS, and CD86 in the colon↓ mRNA expression of IGF-1,and Arg-1 in the colon↑	IRF5 protein↓ p-p38protein↓ SB203580 (speciﬁc inhibitor of p38 MAPK) reduced IRF5 expression. No signiﬁcant change in IRF5 and p-p38 proteinSB203580 (speciﬁc inhibitor of p38MAPK) affected little in IRF5 protein.	^91^
Berberine	F4/80+CD11b+CD16/32+ cells in the colon↓ TNF-a, IL-1b, and IL-6 levels in serum and mRNA expression in the colon↓ IL-10 level in serum and mRNA expression in the colon↑ CD16/32+ cells↓ TNF-a, IL-12, IL-6 level and mRNA expression↓	AKT1 protein and mRNA expression in the colon↑ AKT2 mRNA expression in the colon↓ AKT1 protein and mRNA expression↑ AKT2 mRNA expression in the colon↓ p-p65/NF-kB protein↓ SOCS1 protein↑ siAKT1 reduced CD16/ 32+ cells and SOCS1 promotion and p-p65/NF- kB decline by berberine siSOCS1 inhibited the reduction of p-p65/NF-kB by berberine	^92^
Didymin	F4/80^+^Nos2^+^cells in the colon↓ F4/80^+^CD206^+^cells in the colon↑ Colonic TNF, IL-1b, IL-6, and Nos2 mRNA expression↓ Arg-1, Chil3, Retnla, and IL-10 mRNA expression↑ F4/80^+^Nos2^+^ cells↓ F4/80^+^CD206^+^ cells↑ TNF, IL-1b, IL-6, and Nos2 mRNA expression↓ Arg-1, Chil3, and Retnla mRNA expression↑ No alteration in Arg-1, Chil3, and Retnla mRNA expression↑ IL-10 mRNA expression↑	OCR level↓ Acetyl-CoAlevel↑ Hadhb mRNA expression↑ Hadhb mRNA reverse F4/80^+^Nos2^+^ cells decreased, F4/80^+^CD206^+^cells increased Hadhb mRNA inhibited the reduction of TNF, IL-1b, IL-6, and Nos2 mRNA expression and promoted Arg-1, Chil3, and Retnla mRNA expression	^66^
Dictyophora indusiata polysaccharide	F4/80^+^CD11b^+^ cells in the spleen↓ F4/80^+^TNF-a^+^ cells in the spleen↓ F4/80^+^CD206^+^ cells in the spleen↑ TNF-a, IL-1b, and IL-6 levels and mRNA expression in the colon↓ IL-10 levels and mRNA expression in the colon↑	NLRP3, Bax, and IRF5 Protein in the colon↓ p-STAT3/STAT3 in the colon↓ p-IkBa/IkBa in the colon↓ Bcl-2 and IRF4 protein↑ CD86 in the colon↓	^63^

“↓” Represents a decrease, “↑” represents an increase.

### GinsenosideRg1

One of the primary active components of Panax ginseng, ginsenoside Rg1, has been linked to studies indicating its potential to address various disorders by mitigating inflammation.^93^ Recent research conducted in a mouse model of DSS-induced colitis has shown that ginsenoside Rg1 can significantly alleviate symptoms and the inflammatory response. This effect is achieved by downregulating the production of TNF-a (dose-dependent, *P* < .05),^94^ IL-33, IL-6, and CCL-2 (*P* < .05).^67^ Ginsenoside Rg1 emerges as a crucial regulator of macrophage polarization, promoting the M2 phenotype while inhibiting the M1 phenotype. It also has a modulating effect on the expression of Rock1, RhoA, and Nogo-B proteins in colonic tissues of colitis-afflicted mice. This suggests that the regulation of macrophage phenotype in colitis may be associated with the Nogo-B signaling pathway, similar to Y27632, a specific inhibitor of Rock1.^67^

### Baicalin

One of the active constituents of *Scutellaria baicalensis* Georgi, baicalin, has demonstrated therapeutic benefits in the treatment of IBD.^95,96^ In peritoneal macrophages, baicalin effectively hindered the LPS-induced promotion of the inflammatory macrophage subset M1, leading to a reduction in the M1/M2 ratio. The administration of baicalin alleviated the severity of DSS-induced colitis in mice, potentially through the regulation of IRF4/IRF5 protein expression. Notably, the decline in the M1/M2 ratio induced by baicalin was reversed following the transfection of IRF4 siRNA.^97^

### Toosendanin

Toosendanin (TSN), a triterpenoid present in the bark or fruits of Chinese herbal medicine, has shown promise in modulating macrophage behavior and alleviating symptoms of DSS-induced colitis.^82^ TSN exhibits the ability to reduce the M1 phenotype and the expression of pro-inflammatory cytokines while promoting M2 macrophages. Additionally, it reverses the activation of NLRP3 in colonic macrophages of colitis-afflicted mice. The beneficial effects of TSN are attributed to its activation of the NFE-related factor 2 (Nrf2) pathway, which enhances the antioxidant response by increasing heme oxygenase-1 production and modulating IL-10 production. This dual action influences macrophage phenotype and reinforces the antioxidant response.^98^ These findings suggest that the NLRP3 and Nrf2/HO-1 pathways may be involved in TSN’s control of macrophage modification, attenuating DSS-induced colitis.

### Artemisinin

Artemisinin, the primary compound extracted from *Artemisia annua* L., exhibits a diverse range of actions, including antiviral, antiparasitic, tumor-suppressive, and inflammation-preventive effects.^99^ In Crohn’s disease, artemisinin has been found to enhance M2 macrophages while concurrently reducing the production of pro-inflammatory cytokines. The potential for symptom reduction in colitis is suggested by the upregulation of M2 polarization in colitis tissues through the inhibition of MYD88 and ERK signaling pathways. In a mouse model of DSS, artemisinin demonstrated a significant reduction in MyD88 activation and ERK phosphorylation.^100^

### Tiliroside

Tiliroside, derived from various plants such as linden, rosehip, and strawberry,^91^ has shown promise as a therapeutic agent for UC. It operates by preventing the polarization of M1 macrophages through several mechanisms. Tiliroside facilitates the proteasomal breakdown of HIF-1α, accelerates glycolysis reduction, and downregulates the synthesis of lactate and 2-NBDG in bone marrow-derived macrophages (BMDMs). Additionally, it lowers HIF-1α protein levels, while mRNA expression remains unaffected. Notably, the effectiveness of tiliroside in treating UC diminishes upon the use of clodronate liposomes,^101^ underscoring its role in inhibiting the HIF-1α/glycolysis pathway to reduce M1 macrophages in the context of UC.

### PlatycodinD

Platycodin D (PLD), sourced from the *Platycodon grandiflorum* plant,^102^ was the subject of a study examining its anti-inflammatory properties on LPS-induced RAW264.7 cells and colitis in mice. The research found that PLD effectively mitigated colitis by promoting the M2 phenotype in macrophages. This action was associated with the activation of the PI3K/Akt pathway and the concurrent inhibition of the NF-kB pathway. These changes resulted in a reduction in the nuclear translocation of the p65 subunit and an overexpression of p-PI3K and p-Akt proteins. Importantly, the observed effects were attenuated following the knockdown of AMPK, indicating that the action of PLD is, in part, dependent on AMPK.^103^

### Sulforaphane

Sulforaphane, found in cruciferous vegetables such as broccoli, cabbage, and Brussels sprouts, possesses potent anti-inflammatory and antioxidant properties.^104^ According to research on DSS-induced colitis in mice, sulforaphane treatment greatly improved both the colitis symptoms and the health of the damaged epithelial layer.^105^ Research indicates that sulforaphane stimulates BMDMs to increase the production of interleukin-10 (IL-10). This stimulation prompts macrophages to transition from the M1 to the M2 phenotype in response to lipopolysaccharide (LPS) and interferon-gamma (IFN-g). This process involves the activation of STAT3. There is a possibility that sulforaphane influences macrophage polarization in murine colitis induced by DSS. This influence is strongly associated with IL-10/STAT3 signaling pathways, potentially modulating its effects on M2 phenotypic priority and STAT3 phosphorylation.^92^

### Rhein

Originally utilized in traditional Chinese medicine to address edema, inflammation, and constipation, Rhein is a natural flavonoid compound derived from rhubarb.^106^ Recent findings suggest that Rhein has the potential to alleviate symptoms associated with DSS-induced colitis by influencing macrophage polarization towards the M2 phenotype, known for its anti-inflammatory properties. Experimental data from RAW264.7 cells indicated a significant reduction in the expression of M1 markers and pro-inflammatory mediators following Rhein administration. However, contrasting results were observed for M2 markers. Additionally, researchers noted that Rhein could impede the development and production of IL-1b, a key pro-inflammatory cytokine, in macrophages. This effect was attributed to the inhibition of the Nrf2-dependent redox balance and the Nox2 redox-mediated NLRP3 inflammasome activation.^107^

### Rosmarinicacid

Rosmarinic acid (RA) is obtained from plants in the Lamiaceae family, which includes rosemary, lemon balm, and mint.^108^ A study exploring RA’s impact on DSS-induced colitis in mice revealed promising anti-inflammatory properties, suggesting its potential as a treatment option for UC.^109,110^ The study found that RA significantly reduced the translocation of NF-kB p65 towards the nucleus when the expression of HO-1 protein was decreased. Notably, the NF-kB inhibitor BAY11-7082 did not interfere with RA’s ability to control macrophage differentiation. These findings indicate that RA inhibits HO-1’s capacity to impede the NF-kB pathway, thereby ameliorating experimental colitis by reducing M1 macrophage polarization.^110^

### Genistein

The predominant isoflavonoid present in soy-based products is genistein, renowned for its remarkable anti-inflammatory, anticancer, antioxidant, and antidiabetic properties.^86^ Genistein has recently demonstrated significant efficacy in reducing clinical colitis by targeting macrophage polarization.^87,111,112^ In a recent study, genistein administration led to a notable decrease in M1 macrophages in the spleen mesenteric lymph nodes (MLNs) and colon lamina propria (cLP) of DSS-induced animals, accompanied by an increase in M2 macrophages. Genistein-induced colitis mice exhibited heightened expression of Arg-1 and IL-10 in M2 macrophages compared to those treated with PBS, although the precise mechanism underlying this shift remains unclear.^113^

### Loganin

Loganin, a bioactive iridoid glycoside derived from *Cornus officinalis*, a plant utilized in traditional Chinese medicine, exhibits potent antidepressants, neuropathic protection, and anti-inflammatory properties.^88,114^ Beyond its capabilities in fortifying tight junction proteins that safeguard the intestinal epithelial barrier and curtailing the production of colonic pro-inflammatory cytokines like IL-1b, IL-6, and TNF-a, loganin has demonstrated a notable ability to mitigate the pathological changes associated with DSS-induced colitis.^90^ A study revealed that in colitis mice, M1 macrophages displayed elevated levels of Sirt1, inhibited NF-kB p65 acetylation, and suppressed loganin, effects that were counteracted by the application of the Sirt1 inhibitor Ex527. This suggests that the therapeutic potential of loganin may involve the blockade of the Sirt1/NF-kB pathway.^115^

### Dioscin

Dioscoreanipponica contains a steroid saponin known as dioscin, which has been shown to possess therapeutic properties for colitis treatment.^116^ Dioscin exhibits the ability to inhibit glycolysis and induce the transition of macrophages from the M1 to M2 phenotype. This inhibition extends to the suppression of HIF-1a protein expression, a critical factor in the transcription of inflammatory cytokines and glycolysis-related metabolic genes. The positive impact of dioscin on PPAR-g protein and FAO-related enzymes can be hindered by the mTORC1 signal agonist, while the mTORC2 inhibitor impedes the promotion of M2 macrophages. Dioscin effectively mitigates colitis severity by modulating the mTORC1/HIF-1a and mTORC2/PPAR-g signaling pathways, thereby regulating macrophage polarization and metabolism.^89^ Furthermore, Dioscin triggers the expression of miR-125a-5p, leading to the shift of macrophages toward the M2 phenotype. This shift contributes to the restoration of the intestinal epithelial barrier function, ultimately facilitating clinical improvement in colitis conditions.^117^

### Lupeol

Luteol, a triterpenoid molecule found in various natural plants like *Albizia lebbeck* and *Alnus glutinosa*, possesses distinctive bioactivity.^118^ Experimental findings suggest that lupeol, derived from luteol, imparts protective benefits against colitis in mice by influencing the NF-kB signaling in IECs. This modulation leads to a cessation of signaling, and macrophages, favoring the M2 phenotype, undergo modifications that result in reduced inflammatory responses.^119,120^ Lupeol’s impact is notable in its ability to decrease IRF5, a transcription factor associated with M1 macrophages, particularly when induced by LPS and IFN-g with exposure to GM-CSF. Interestingly, this effect is not observed in M2 macrophages, indicating a specific modulation of signaling pathways. Additionally, lupeol reduces the phosphorylation of p38 MAPK in M1 macrophages, a process that can be reversed by employing a p38 MAPK inhibitor.^119^ The mechanism by which lupeol facilitates the transition of M1 macrophages towards the M2 phenotype involves the potential blockage of IRF5 through a specific receptor and downstream signaling pathway, such as p38 MAPK. This suggests a targeted and intricate signaling pathway modulation by lupeol in regulating macrophage behavior.

### Berberine

Berberine, an isoquinoline alkaloid abundant in the root of *Coptis chinensis*,^121^ has proven efficacy in colitis treatment. This effectiveness is attributed to its multifaceted actions, including the inhibition of the IFN-g and JAK2/STAT3 signaling pathways, leading to a reduction in inflammatory responses.^122^ Moreover, berberine influences the Wnt/β-catenin pathway, crucial for maintaining immunological homeostasis in the intestinal mucosa.^123^ Additionally, it plays a role in curtailing the generation of inflammatory cytokines by inhibiting the MAPK and NF-kB signaling pathways.^124^ Recently, Yunxin et al. reported that Berberine can correct macrophage polarization imbalance by inhibiting M1 phenotype differentiation and preventing colitis development by upregulating the AKT1 pathway, SOCS1 protein expression, and decreasing NF-kB phosphorylation. AKT1 small interfering RNA transfection neutralizes its effects on M1 polarization and related pro-inflammatory cytokines, IL-10, and TNF-a indicating its inhibitory activity depends on the AKT1/SOCS1/NF-kB signaling pathway.^125^

### Didymin

Didymin, a dietary glycoside found in citrus fruits like mandarin, bergamot, orange, Origanum, and Vulgare Duanxueliu, is known for its antioxidant properties.^85^ Didymin can reduce colitis in mice by targeting macrophage polarization to the M2 phenotype. It decreased M1 macrophage proportion and increased M2 in the colon. Mice injected with exogenous M1 macrophages showed increased sensitivity to DSS. However, didymin management reduced colitis severity before injection. Didymin resisted M1 macrophage polarization but did not alter M2 macrophages or expression of Arg-1, Chil3, and Retnla. The effect of didymin on ameliorating colitis depends on M1 macrophage transformation towards M2. The macrophage phenotype of didymin is influenced by enhanced fatty acid oxidation (FAO) by enhancing the expression of Hadhb.^83^ A new study hypothesized that the enhancement of FAO produced by promoting the expression of Hadhb is how the macrophage phenotypic modulation of didymin is expressed.

### Dictyophora indusiata polysaccharide

A powerful antioxidant and anti-inflammatory compound called Dictyophora indusiata polysaccharide (DIP) has been identified among this famous edible fungus owing to its daintiness and nutritional diversity.^126,127^ Dictyophora indusiata polysaccharide may reduce colitis severity in mice with DSS by alleviating oxidative stress, modulating macrophage polarization, and recovering gut microbiota activity and epithelial integrity.^128–130^ Dictyophora indusiata polysaccharide treatment reduced M1 macrophage polarization and promoted M2 phenotype in mice orally administered DSS. Inhibition of CD86-marked macrophages in the colon signifies that IL-6, IL-1b, and TNF-a expression are dysregulated and that IL-10 secretion is elevated. DIP also downregulated NF-kB, STAT3, and NLRP3 signaling pathways in the colon, possibly affecting macrophage polarization balance.^128^ The biological function of polysaccharides is strongly connected with their spatial structure, so when they are broken down into monosaccharides or oligosaccharides, their effectiveness is considerably diminished as well as eliminated.

## Future Prospective and Conclusion

Ulcerative colitis patients may require lifelong medication to prevent recurrence, lower the risk of colon cancer, and enhance their overall quality of life. However, clinical remission patients face an increased risk due to inadequate adherence to prescribed medications.^131–133^ Currently, 5-aminosalicylic acid (5-ASA) drugs are the first choice treatment in the clinic for mild to moderate UC. These medications can be given as pills, enemas, or oral preparations.^78^ Corticosteroids^79^ can be prescribed to patients who do not respond to or do not reach remission with 5-ASA medications. However, it is not recommended to use glucocorticoids to maintain remission due to their adverse effects risks, and lack of long-term effectiveness.^80^ Patients with moderate to severe colitis should be prescribed thiopurines or biologic medicines, or a combination of the 2. It is important to closely monitor patients using these medications over an extended period to help prevent side effects such as lymphoproliferative diseases.^134,135^ When endoscopic methods fail to remove UC-associated unfavorable lesions, significant bleeding that cannot be controlled, or perforation occurs, surgical intervention may be necessary.^81^ Nature-derived phytochemicals, which are easy to find, have been shown to have a wide range of biological functions, as well as being relatively safe and highly effective, which makes them especially useful for fighting against disease.^82–84^ Exploring alternative strategies, herbal compounds, particularly flavonoids, emerge as a promising, low-risk therapeutic option. Flavonoids have shown potential in altering immune cell numbers, disrupting cellular signaling pathways, controlling inflammatory cytokines and intestinal flora, and preserving the immunological barrier of the intestinal mucosa.^136^ The persistent and recurrent nature of UC, classified as a contemporary refractory illness, significantly impacts patients’ quality of life, leading to symptoms such as persistent haematochezia and stomach.^81^ Studies emphasize the pivotal role of macrophage polarization imbalance in UC development, with a focus on shifting macrophages towards the anti-inflammatory M2 phenotype.^44,59,68,137^

Natural products, rich in bioactivities and nutrients, stand at the forefront of future medicine research. Several phytochemicals, including didymin, genistein, and loganin, have demonstrated potential in treating experimental colitis by modifying macrophage polarization.^83,113,115^ The chemical components of flavonoids, polyphenols, alkaloids, and terpenoids play a crucial role in controlling various signaling processes, offering potential therapeutic avenues.^83,89,115^ Inflammatory bowel disease, a common condition, activates NF-kB pathways, negatively influencing disease progression. Researchers are exploring phytochemicals from medicinal plants to regulate NF-kB activation, decreasing inflammatory mediators while increasing the expression of barrier proteins. However, the therapeutic efficacy of these phytochemicals in UC patients remains to be investigated.

Herbal remedies for chronic gastrointestinal issues, like UC, represent a widely utilized approach globally. Physicians should provide evidence-based information and conduct clinical double-blind trials to validate the effectiveness of these treatments. Combining flavonoids with other drugs for comprehensive treatment shows promise, and the development of specific, highly effective flavonoid pharmaceuticals is feasible. Further research is needed to understand the effects of phytochemicals on macrophage polarization, along with considerations of toxicity and safety. Investigating the effectiveness and safety of naturally occurring NF-kB regulators in treating IBD is also ongoing. Utilizing natural substances targeting the Nrf2 pathway for IBD treatment presents significant potential for creating small-molecule medicines or microorganisms that activate the Nrf2/ARE pathway. Controlling and rationalizing flavonoid natural product research is crucial to enhance therapeutic outcomes and facilitate the development of novel medications for treating UC. According to the above results, phytochemicals may help ease the symptoms of UC by changing the polarization of macrophages. Nevertheless, there has been a lack of studies on their effectiveness in treating UC in humans, as all previous studies have relied on animal models. Also, additional information is needed to clarify the regulatory processes and possible toxicity of these phytochemicals that control macrophage polarization.

## Data Availability

No new data were created or analyzed in this study. Data sharing is not applicable to this article.
